# Artificial intelligence in age-related macular degeneration: state of the art and recent updates

**DOI:** 10.1186/s12886-024-03381-1

**Published:** 2024-03-15

**Authors:** Emanuele Crincoli, Riccardo Sacconi, Lea Querques, Giuseppe Querques

**Affiliations:** 1 Ophthalmology Unit, “Fondazione Policlinico Universitario A. Gemelli IRCCS”, Rome, Italy; 2 Department of Ophthalmology, University Vita-Salute IRCCS San Raffaele Scientific Institute, Via Olgettina, 60, 20132 Milan, Italy

**Keywords:** Artificial intelligence, Age-related macular degeneration, AMD, Prediction, Deep learning, Support vector machine, OCT, Treatment

## Abstract

Age related macular degeneration (AMD) represents a leading cause of vision loss and it is expected to affect 288 million people by 2040. During the last decade, machine learning technologies have shown great potential to revolutionize clinical management of AMD and support research for a better understanding of the disease. The aim of this review is to provide a panoramic description of all the applications of AI to AMD management and screening that have been analyzed in recent past literature. Deep learning (DL) can be effectively used to diagnose AMD, to predict short term risk of exudation and need for injections within the next 2 years. Moreover, DL technology has the potential to customize anti-VEGF treatment choice with a higher accuracy than expert human experts. In addition, accurate prediction of VA response to treatment can be provided to the patients with the use of ML models, which could considerably increase patients’ compliance to treatment in favorable cases. Lastly, AI, especially in the form of DL, can effectively predict conversion to GA in 12 months and also suggest new biomarkers of conversion with an innovative reverse engineering approach.

## Introduction

Artificial intelligence (AI) has already revolutionized our way of living, and it is destinated to induce even more profound changes in many sectors of modern society. Among them, healthcare, and especially imaging based subspecialties such as ophthalmology, have the highest potential to benefit from integration of AI in everyday clinical setting. Two main approaches can be distinguished: Feature learning (FL) and Deep Learning (DL). While in the first one classification is based on predetermined variables, the deep learning approach is based on the ability of neural networks to identify differences between cases. The unknown nature of this differences is at the origin of the so-called black box effect, consisting in the uncertainty over the reliability of the classification performed by the model due to the lack of information about guiding elements. To solve this problem, many strategies have been attempted to explain and visualize the decisional mechanism hidden within each model (explainability in AI). DL models are versatile software that can be used for different tasks. In imaging research, DL can be used for two main purposes: segmentation of structures or classification of cases (or a combination of both). Both FL and DL models need to be validated on a separate population after the training phase is completed, a process that goes by the name of testing (or external validation). The generalizability of a model defines the applicability of the model to the general population. Accurate models can support clinical management of eye diseases, especially high prevalence ones. In particular, the use of machine learning (ML) techniques has been tested in ophthalmology in the fields of screening, diagnosis, clinical decision-making and prediction of prognosis with promising results. Age related macular degeneration (AMD) is a multifactorial disorder representing a leading cause of vision loss and it is expected to affect 288 million people by 2040 [1]. The aim of this review is to provide a panoramic description of all the applications of AI to AMD management and screening that have been analyzed in recent past literature.

### Diagnosis of AMD

#### Predicting incipient AMD

An effort towards a better understanding of the diseases on a genetic point of view using machine learning methods was done by Yan et [2]., that compared the performance of 4 different machine learning techniques (neural network, lasso regression, support vector machine, and random forest) in assessing the risk of AMD on a database of more than 32,000 caucasian individuals. The analysis was also meant to assess feasibility of prediction of AMD risk using genome analysis. All models reached around 0.80 area under the curve (AUC) when tested on data from the same biobank and an AUC of around 0.70 when tested on a different biobank.

An interesting study from Lee et al. [3] used a deep learning model trained on fovea crossing optical coherence tomography (OCT) images to identify OCT biomarkers of delayed rod-mediated dark adaptation (RMDA), which is a known functional biomarker for incipient AMD. The model identified hyporeflective outer retinal bands on macular spectral domain (SD) OCT associated with delayed RMDA with an acceptable mean absolute error (MAE).

#### AMD automatic diagnosis

Several algorithms have been trained for automatic detection of AMD on various imaging modalities. Many of them were based on the segmentation and counting of drusen and drusen-like deposits and were aimed to identification of the disease at its early stage. Yildirim et al. [4] trained and tested a U-Net deep learning (DL) segmenter to the identification of early AMD OCT biomarkers. The model obtained very good accuracy proving its potential in facilitating AMD screening with the contribution of automatic patient selection. Morelle et al. [5] reported the results of an OCT segmenter based on DL technology that was able to quantify drusen load with excellent accuracy based on layer positions, achieving an exceptional correlation between drusen volumes estimated with this method and two expert human readers, and increasing the Dice score compared to a previous state-of-the-art method [6]. Other authors [7] proposed a DL framework to automatically distinguish drusen from reticular pseudodrusen (RPD) that was meant to prompt further understanding of RPD as a separate entity from drusen in both research and clinical settings. The model achieved > 90% accuracy in classification and segmentation, which was similar to human experts’ performance. Accurate identification of RPD was also confirmed by different authors [8]. 

Saha et al. [9] tested for AMD diagnostic performance different DL algorithms pretrained for detection and classification of hyperreflective foci, hyporeflective foci within the drusen, and subretinal drusenoid deposits from OCT B-scans. An overall accuracy of 87% for identifying the presence of early AMD biomarkers was achieved.

Despite the good diagnostic results obtained with drusen identification, as highlighted by Thakoor et al. [10], the best diagnostic performance was obtained by DL models using multimodal imaging as input, in particular when OCT B scan and OCT angiography (OCTA) acquisitions were provided to the software. Other authors demonstrated good results with a combination of OCT B scan and color fundus imaging [11]. 

In a metanalysis from Leng et al. [12], the type of AMD and the architecture of the DL model appeared to be the main reasons for heterogeneity of the results obtained in AMD diagnostic performance. In particular ResNet architecture was identified as the most suitable DL design for optimization of the task. In alternative, architectures with < 10 layers might be preferable to overcomplicated models not addressing the problem of vanishing gradients (which is brilliantly managed in ResNet).

FDA recently approved iPredict AMD, a DL screening tool available on the market that can detect referrable AMD with 88% accuracy. This tool can also predict individual risk score for development of late AMD within 1 and 2 years [13]. 

#### Predicting progression to late stage AMD and identifying late stage biomarkers

Several studies have proven good performance of DL in segmentation and quantification of subretinal and intraretinal fluid in exudative AMD [14–18]. 

Identification of macular atrophy for automatic diagnosis of advanced AMD has also been tested. Wei et al. [19] demonstrated high performance of a DL model in identification of 6 imaging features associated to macular atrophy in AMD patients. The selected features were the presence of interrupted outer retina and interrupted retinal pigmented epithelium (RPE), the absence of outer retina and RPE, and the presence of hypertransmission < or > 250 μm.

Other authors [20] presented a highly performing fully automated algorithm segmenting Retinal Pigment Epithelial and Outer Retinal Atrophy (RORA) in dry AMD on macular OCT. The results of the segmenter turned out to be comparable to the ones of expert human graders.

Assessment of the risk of progression from an uncomplicated form of AMD to a late-stage AMD (either neovascular or atrophic) was also attempted.

Schmidt-Erfurth et al. [21] elaborated a ML model using a combination of demographic, and genetic input features as well as automated volumetric segmentation of outer neurosensory layers and retinal pigment epithelium, drusen, and hyperreflective foci by spectral domain-OCT image analysis with the aim of assessing the risk of conversion to advanced AMD. While the model obtained good results in prediction GA development (AUC 0.80), macular neovascularization (MNV) development was not as reliably predicted (AUC 0.68). Bhuiyan et al. [13] used color fundus photographs of the patients from the AREDS study to train a DL model for automatic recognition of the stage of the disease (early/none vs. intermediate/late), obtaining a 99.2% accuracy. They then used this information combined with sociodemographic data to train a feature learning model to assess the risk of conversion towards a neovascular AMD or geographic atrophy (GA) during the follow up. The prediction model for a 2-year incident late AMD (any) achieved 86.36% accuracy, with significantly lower performance when specific type of late-AMD (either wet or dry) was to be detected. Burlina et al. [22] also discussed how DL technology could not only classify AMD cases with the 9-step AREDS severity scale as accurately as expert human graders, but also provide reliable 5-years prediction of evolution to late-stage disease.

### Neovascular AMD

The risk of conversion to the neovascular form of the disease and exudation has also been evaluated using ML technologies. Benerjee et al. [23] proposed a Deep sequence approach combining imaging features, demographic, and visual factors, with a recursive neural network (RNN) model in the same platform to predict the risk of exudation in non-exudative AMD eyes in the short term (within 3 months) and long term (within 21 months). In particular, results in short term prediction appeared to have high generalizability when tested on an external dataset.

#### Prediction of the burden of treatment

The first study to predict anti vascular endothelial growth factor (VEGF) treatment needs in AMD was published in 2017 by Bogunovic et al. [24]. The authors demonstrated high accuracy of a model integrating a combination of baseline, 1-month and 2-months OCT features, initial best corrected visual acuity (BCVA) and demographic characteristics in predicting the burden of intravitreal injections (IVIs) of ranibizumab needed within a 2 years follow up in a pro re nata (PRN) regimen (data from the HARBOR study). Classification of low (≤ 5) and high (≥ 16) treatment requirement subgroups demonstrated around 75% accuracy, with the best prediction obtained for values at 2 months. Subretinal fluid volume in the central 3 mm was identified as the most relevant feature for prediction. Recently, Chandra et al. [25] used data from the Comparison of AMD Treatments Trials (CATT) to investigate the performance of 3 different feature learning (ML) models in prediction of the number of IVI needed in a pro re nata (PRN) regimen after the loading phase in the first 2 years of treatment. The outcome was evaluated both as total number of injections in two year and in a categorial manner, identifying patients who received few (≤ 8) or many (≥ 19) injections within the same follow up time. According to their results, the best performing model was the SVM, with an area under the curve (AUC) of around 0.80 in binary prediction of few/many injections. Important features included fluid in optical coherence tomography (intraretinal, subretinal, or sub-RPE), lesion characteristics, and treatment trajectory in the first three months. Baseline lesion characteristics included macular neovascularization (MNV) lesion area, lesion location (subfoveal or non-subfoveal), lesion composition (considering lesions such as MNV, hemorrhage, blocked fluorescence, and serous retinal pigment epithelial detachment), and lesion type (occult only, minimally classic, or predominantly classic).

Pfau et al. [26] proposed a probabilistic forecasting of the number of injections needed in a real life setting with 1 year follow up, demonstrating a mean absolute error (MAE) in prediction of the burden of anti-VEGF treatment frequency of around 2.6 injections /year.with the proposed model.

As concerns treat and extend (TE) regimen, the potential of feature learning (in particular random forest architecture) to predict high (< 5 weeks interval) or low (> 10 weeks interval) treatment demand in AMD, retinal vein occlusion (RVO) and diabetic macular edema (DME) was analyzed by Gallardo et al. [27] The AMD-trained models yielded an AUCs around 0.80 for both low and high demand. Even more importantly, this study revealed that it is possible to predict low demand reasonably well at the first visit, before the first injection.

Deep learning technology was also tested in its ability to predict the need for treatment. Romo-Bucheli et al. [28] proposed a DL model including DenseNet [29] structure and a RNN (trainable end-to-end) architecture to predict IVIs burden during a PRN regimen. The model predicted number of received injections with a concordance index of 0.7 and demonstrated a 0.85 (0.81) AUC in detecting the patients with low vs. high treatment requirements.

Lastly, Hwang et al. [30] demonstrated how a DL algorithm trained on 35,000 OCT images could learn to provide correct treatment indications, which is particularly interesting in primary care and telemedicine settings.

#### Predicting the choice of treatment and treatment results

In a 2023 publication, Moon et al. [31] reported the results of a DL model conceived to guide the clinician in the choice of treatment (aflibercept vs. ranibizumab). The model was trained on OCT images and its architecture was based on an attention generative adversarial network (GAN) model. They highlighted how the AI model predicted anti-VEGF agent-specific short-term treatment outcomes with higher sensitivity than both highly and less experienced human examiners, thus proving the potential advantages of its use in everyday clinical practice.

Machine learning technology may also help predict the visual outcomes of anti VEGF treatment. The performance of 5 different feature learning algorithms to this task was tested, showing the Lasso protocol as the best performing [32]. This model obtained a 5-letters mean absolute error in 3 months prediction and 8 letters mean absolute error in 12 months prediction. The authors discussed how a similar tool might increase compliance to treatment, especially when 12 months results are prospected to the patient. Fu et al. [33] obtained even higher performance in post-treatment VA prediction using DL technology, particularly in the form of an OCT segmenter providing biomarkers quantification and changes registration during the course of the treatement.

### Geographic atrophy

Quantification of GA is extremely important for disease monitoring, analysis of risk factors for progression and evaluation of clinical endpoints. Moreover, accurate, repeatable and easy methods for GA area calculation would also help investigating structure-function correlation and elucidating pathophysiological mechanisms of disease development and progression. Balaskas et al. [34] demonstrated feasibility of residual visual acuity prediction using a random forest model trained with DL-segmented GA biomarkers on OCT images. The status of the foveal region (46.5%) and RPE-loss (31.1%) had greatest predictive importance for VA. For low luminance VA, however, non-foveal regions (74.5%) and photoreceptors’ degeneration (38.9%) were most important. Other authors demonstrated accurate segmentation of GA on fundus autofluorescence imaging [35]. 

#### Conversion to GA and GA progression

With an interesting and innovative concept of AI use in ophthalmology [36], Wang et al. [37] proposed a different approach to biomarkers identification, which was based on reverse engineering technology. In fact, the model was intended to identify new potential biomarkers of GA with the help of explainability methods. The reconstructions consistently highlighted that large foveal drusen and drusen clusters with or without mixed hyper-reflective focus lesion on baseline OCT were often present in eyes experiencing conversion to GA after 12 months.

Gigon et al. [38] proposed a DL method for automatic retinal pigment epithelial and outer retinal atrophy (RORA) progression prediction. The proposed software was based on enface multiple reconstructions of the status of the outer retina and provided continuous-time output. It was used to compute atrophy risk maps, which indicate time-to-RORA-conversion, that represents a novel and clinically relevant way of representing disease progression.

### New perspectives

Natural language processing models have been shown to provide satisfactory responses to medical queries posed by AMD patients. In a recent study, Johnson et al. showed how Chat-Generative Pre-Trained Transformer(Chat-GTP) generated responses that were judged with a mean score of “almost completely correct” and a mean score of “complete and comprehensive” as concerns respectively accuracy and completeness [39]. The use of Generative adversarial networks (GANs)(consisting in two competing types of deep neural networks, including a generator and a discriminator), although still in its early phases, is showing promising potential applications in ophthalmology as described in an interesting review from You et al. These include, conversion, artifact removal, denoising and database expansion, which could be applied to AMD imaging to aid diagnosis and interpretation [40]. 

## Conclusions

During the last decade, machine learning technologies have shown great potential to revolutionize clinical management of AMD and support research for a better understanding of the disease.

DL based diagnosis of AMD is easier when multimodal imaging serves as input (OCTA and OCT B scan), even though the approach based on drusen identification only may lead to satisfactory results with lower economic burden. The use of ResNet architecture is advisable to optimize diagnostic performance. Accurate diagnosis of referrable AMD and prediction of risk of development of advanced form of the disease within 1 and 2 years can be provided by a commercially available software recently approved by FDA to this scope (iPredict AMD, iHealthScreen).

As concerns neovascular AMD, short term risk of exudation may be effectively predicted using a combination of imaging and demographic and clinical information. Machine learning can also help predict the need for injections within the next 2 years. This can be achieved with both feature learning methos (among which the SVM technology might be the most suitable method) and DL methods. Prediction of few IVIs needed is particularly proficient and can be accurately predicted very early during the treatment (ideally before the first injection). The most relevant feature appears to be subretinal fluid volume in the central 3 mm, even though in general the unsupervised approach used by the DL methods may obtain better results in this type of task. Moreover, DL technology has the potential to customize treatment choice with a higher accuracy than expert human graders. In addition, accurate prediction of VA response to treatment can be provided to the patients with the use of ML models, which could considerably increase patients’ compliance to treatment in favorable cases. Considering the positive results, there is a good chance that in the next future treatment interval and choice for wet AMD will be supported by AI technology. In order to make the best out of this additional tool, this revolution will certainly require economic evaluation and adjustments in the procedures for management of wet AMD patients in the real life.

Lastly, AI, especially in the form of DL, can effectively predict conversion to GA in 12 months and also suggest new biomarkers of conversion with an innovative reverse engeneering approach.

## Data Availability

No datasets were generated or analysed during the current study.

## Abbreviations

AI: artificial intelligence
AMD: age-related macular degeneration
AUC: area under the curve
BCVA: best corrected visual acuity
DL: deep learning
DME: diabetic macular edema
GA: geographic atrophy
IVI: intravitreal injection
MAE: Mean absolute error
ML: machine learning
MNV: macular neovascularization
OCT: optical coherence tomography
OCTA: optical coherence tomography angiography
PRN: pro re nata
RF: random forest
RMDA: rod-mediated dark adaptation
RORA: Retinal Pigment Epithelial and Outer Retinal Atrophy
RPD: reticular pseudodrusen
RPE: retinal pigmented epithelium
RNN: recursive neural network
RVO: retinal vein occlusion
SD: spectral domain
SVM: support-vector machine
TE: treat and extend
VEGF: vascular endothelial growth factor
